# Design Study for Automatic Production Line of a Sub-Assemblies of New Generation Car Body Structures Compliant with the “Industry 4.0” Concept

**DOI:** 10.3390/s21072434

**Published:** 2021-04-01

**Authors:** Ireneusz Wrobel, Marcin Sidzina

**Affiliations:** Faculty of Mechanical Engineering and Computer Science, University of Bielsko-Biala, 43-309 Bielsko-Biala, Poland; msidzina@ath.bielsko.pl

**Keywords:** hot forming, 22MnB5, car body, Industry 4.0

## Abstract

A design study of automatic line-to-production of a new generation of car body structures compliant with the Industry 4.0 concept is described in this paper. The line is based on the hot-stamping technology of components of a car body structure from 22MnB5 steel sheets. Additional modules of the designed production line are: laser-trimming station, station to completion (kitting-up), and spot-welding station of the subassemblies. Technical requirements to be complied with by such line and scheme of exchange of information between modules of the line were defined. The conclusions were formulated.

## 1. Introduction

Manufacturing systems used in the automotive industry have to meet challenges of the 21st century. These challenges can comprise, among others: demographic changes in the society, permanent variability of technological processes and growing complexity of the products, standardization of the manufacturing processes, short operational life of the products, high flexibility of the manufacturing lines, implementation of information technology and communication technology [1,2] between cooperating smart factories, autonomy of the manufacturing lines [3,4,5], self-controlling ability of operation of the lines [6,7], unequivocal traceability of quality and parameters of the manufactured products [8], and new properties of materials used to manufacture the products [9,10]. The fourth industrial revolution is the answer to this challenge. This belongs to topics of frequent discussions between engineers and scientists working in the automotive industry [11]. The term “Industry 4.0” describes a vision of intelligent and automated factories with mutually interconnected manufacturing systems, manufactured products and customers. Manufacturing systems compliant with Industry 4.0 are modular and they closely cooperate with each other, permanently share information and data, operate in an autonomous way, and independently control technical conditions of machinery and devices; these systems are flexible and prepared for quick changes in the manufacturing profile and short operational life of the products. Manufactured products carry with them information on parameters of their production and quality [12,13].

Development of the automotive industry is driven by new requirements connected with protection of the environment [14]. This is related to restricted emissions of exhaust gases from vehicles powered by traditional driving systems and very fast development of electric vehicles [15,16]. These issues belong to one of the most important technical problems that the automotive industry has to face nowadays. To meet such demands, the automotive industry has focused on development of lightweight body structures using materials characterized by a high strength-to-weight ratio.

Car body structures of new-generation passenger cars should feature possibly the lowest mass while maintaining all safety standards in force. Certain regularity can be observed, as new models of popular car brands are lighter than the previous models by at least 20%. This tendency has increased even more in electric car designs, where mass of the battery needs to be compensated for. Reduction of mass of the car body is possible owing to usage of new ultradurable materials and new manufacturing technologies, and joining technologies of car body elements [17].

Currently, the automotive industry is facing big challenges in terms of sustainable development in areas of reduction of CO_2_ emissions, energy savings and efficient use of natural resources. This is connected with use of very high-strength steel for production of car body structures. The drawpieces produced in the hot stamping process are weight-optimized, which is connected to energy savings in the course of operation of the cars [18,19].

The hot stamping from the 22MnB5 steel belongs to one of the technologies enabling production of car body components featuring very high mechanical properties (strength limit 1300 to 1900 MPa, hardness 400 to 500 HV) [20]. Joining the components together belongs to important manufacturing stages of the subassemblies. In cases of joined components made of the same material (e.g., from steel), the most often used components are joined together using electrofusion welding, arc welding or laser welding [21,22,23,24]. In cases of the components made of various types of materials (steel–aluminum, steel–composite), different joining methods are used: clinching, self-pierce riveting or bonding [25,26]. All described manufacturing processes belong to very advanced ones and are accomplished on automated, flexible and robotic production lines. Big automotive consortiums require their subsuppliers to use flexible production lines, allowing prompt change of manufactured assortment—which are communicated with car body assembly lines using informatics systems—to have access to qualitative information on produced subassemblies, and to have information about selected technological parameters of the production process of each subassembly [27,28,29]. A design study of manufacturing line to production of car body subassemblies, which will comprise a hot stamping module, automated station-to-laser trimming of the drawpieces, and automatic welding station of the drawpieces, will be presented in this paper. The subassembly consisting of several cold-stamped drawpieces and one hot-stamped drawpiece is the end product of such a production line. The proposed production line is set to operate in one from automotive companies in Poland.

The novelty of the proposed solution is the possibility of constant monitoring of the quality of each component produced by the production line, and the system for real-time controlling and diagnosing the condition of the machines and production equipment. This will enable quick identification of quality problems of manufactured products, will prolong the time of trouble-free operation of the machines operated on the line, and will reduce the manufacturing costs. A manufacturer of the car bodies will have access to the database, where information on quality parameters of each manufactured product will be recorded. This will enable constant monitoring of deliveries and improvement of quality of the manufactured car bodies.

## 2. Industry 4.0 in Production of Hot-Stamped Components of Car Body Structure

Implementation of the Industry 4.0 assumptions in the hot-stamping process of car body components was presented by Gonzalez et al. [30]. The developed system for acquisition, storage, processing and analysis of the large amount of data coming from machines and devices accomplishing production on automated lines enables quick identification of sources of technological problems occurring on the production line, improvement of production capacity, continuous controlling of the manufacturing process with real-time notification of operating parameters of the line, minimization of a down times, and so on. A similar solution from the area of monitoring parameters of the processes and parameters of selected machines installed on the line to the hot stamping is presented by authors of the study [31]. The proposed system collects data from the press, furnace, elements of automation, thermo-vision cameras and other sensors, processes them in an appropriate way to assure further drawing conclusions, and saves them to the database. Owing to this, it is possible to take suitable preventive measures when problems in the production process, or problems connected with technical conditions of machines installed in the line can occur.

It should be emphasized that owing to the application of the Industry 4.0 concept in the production of automotive subassemblies, the production lines will be more flexible and efficient, material flows will be optimized, and efficiency of the resources will increase. This will result in moving away from mass production, which is a source of waste of resources, energy and environmental pollution [32].

## 3. Requirements for the Designed Production Line

Comprehensive and unequivocal identification of manufactured subassembly, taking into account its qualitative parameters (shape–dimensional accuracy and mechanical parameters) is the main objective of launching the new production concept based on idea of Industry 4.0. Each subassembly will be marked with a unique number associating its qualitative and manufacturing parameters (e.g., temperature in chamber of the furnace, temperature of cooling medium, feed rate of laser cutting, welding current intensity). Elaboration of the system to autonomous control of conditions of the machines and devices working in the production cell is the additional objective of the undertaken activities.

Designed production line should be flexible, enabling production of different subassemblies after replacement of specialized tooling and control software.

The production line was divided into three modules:

- module to production of hot-stamped drawpieces,
- laser trimming station,
- station to completion (kitting-up) and welding of the subassemblies.

**Figure 1:**
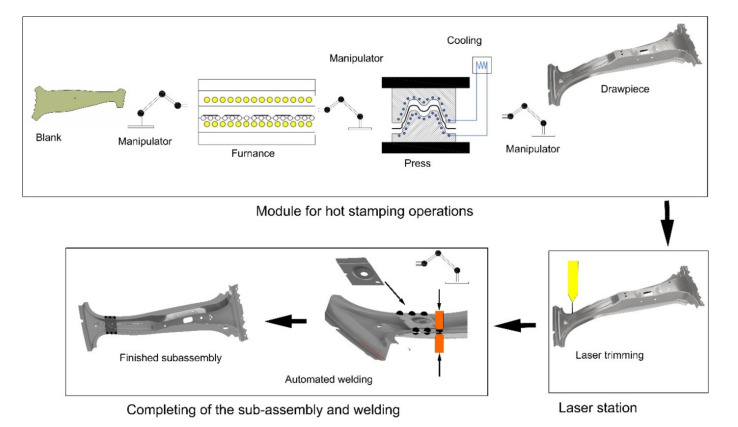
Scheme of the automatic line to production of car body subassemblies.

A scheme of the automatic line to production of subassemblies made of the 22MnB5 manganese–boron steel destined to the hot stamping is reported in Figure 1.

A central control system is responsible for the controlling, and is responsible for flow and analysis of the information on the production line. The system consists of the following three elements:

1. Integrated system to control the production line, having the following tasks to perform:
  (a) control of automatic manufacturing processes (hot stamping, laser trimming and kitting-up with welding),
2. System to control technological and qualitative parameters of produced subassemblies and record them in the database together with the unique number of the subassembly, which shall be responsible for:
  (a) read-out of the technological parameters in all operations of the technological process,
  (b) quality control of the production (verification of shape and dimensions, control of mechanical parameters),
  (c) continuous monitoring of the technological and qualitative parameters on all stages of the production,
3. System for monitoring and auto-diagnosis of conditions of individual machines installed in the line, which shall be responsible for:
  (a) read-out of signals from sensors installed on monitored devices and machines, processing the signals and making conclusions,
  (b) automatic responsiveness of the system, which monitors conditions of machines in the line, to detected irregularities.

All three of the above-specified systems are governed by a single integrated information system, which is responsible for acquisition, processing and analyzing of signals coming from the sensors installed in the line. The signals can be divided into:

(a) control-measurement signals: responsible for the most important technological parameters of individual technological operations accomplished in the line (for instance: temperature in the furnace, time of soaking, etc.),
(b) signals responsible for diagnostics and conditions of the machines installed in the line (for instance: amplitude of vibrations and temperature of hydraulic pumps, temperature of housing of the furnace, etc.),
(c) signals coming from the system controlling components of the automated line (for instance: signals from safety devices, signals analyzing trajectory of the robots, etc.).

**Figure 2:**
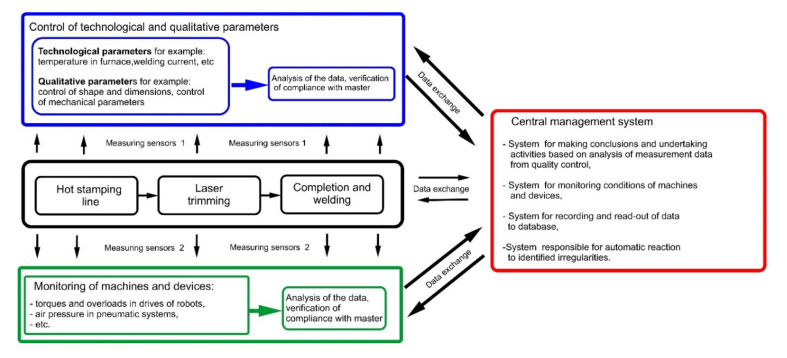
Scheme of exchanging information in the production line, compliant with concept of Industry 4.0.

Figure 2 presents a scheme depicting the constructed system.

A special structure of the control system of the production line discussed here was proposed, based on devices installed in the production line and equipped with their own independent control systems. Integration of the signals coming from individual devices was the main issue faced by designers of the system. The integration was solved at a central level using the main PLC controller, called “Master”. The task of this controller is to collect signals from all devices and to decide which tasks are to be performed at a given time, for example, which movement program should be accomplished by individual robots. The system is so complicated that it connects many different actuators, communicating with each other through various communication protocols, including Profinet, Profibus DP, OPC/UA. The above task is not a trivial one, because it requires real-time operation. For this purpose, the PLC controller with distributed input/output systems was used. Centralization of the system has allowed collection of vectors of the states of all input and output signals, and in the future it will allow for the redundancy of the central unit. Nevertheless, in the current state, computing power of the central unit allows for real-time control of the production line and the digital twin of the line, designed using the Tecnomatix Process Simulate software, (Siemens, Munich, Germany) [33]. A mechanism for comparison of vectors of the state of the devices between the real line and the digital twin is being built. The objective of the above is to find errors from differences in vectors of the states of selected variables. This analysis will allow for quick verification of states of the signals from the production line. It can be assumed that the digital twin, initially in a simplified form, will allow for prompt localization of signals of anomalies and will allow for development of patterns of the errors, which will be analyzed by a special analytical system.

## 4. Implementation of Automated Line to Production of Car Body Subassemblies

The designed automated production line should be able to produce the subassemblies according to requirements of the customers, mainly in terms of quality. Actually, such a type of production is carried out on different, not interconnected, working stations. These are also arranged in various workshops assigned to different production departments (hot-stamping workshop, spot-welding workshop, welding workshop). The hot-stamping module works independently from the operation of the laser trimming device. Station-to-completion and spot welding are also independent. The objective of this new production concept is to combine the hot-stamping production module, the laser-trimming station, and the completion station into a single automated production line. Knowledge, so called “know-how”, and the best practices concerning individual technologies as used in the individual modules of the production line were taken into consideration in designing such a line. Such knowledge is scattered among engineers, but also among workers, who operate specific devices. This knowledge has been worked out and confirmed in practice during many years of work; taking it into account is indispensable to achieve assumed targets.

To gather this knowledge, appropriate surveys were used and a number of interviews were conducted with engineering staff and employees working in specific positions (hot stamping, laser trimming, as well as completion and welding).

After processing results of the performed surveys and interviews, gathered knowledge was suitably cataloged and divided into three groups, destined for:

- automatic system, controlling the production line (all its elements); more than 40 parameters have been extracted that will be taken into account in the course of its designing. The most important of these parameters are: time of soaking of the blank in the furnace, transfer time of the blank from the furnace to the stamping die, control of electrical power of the laser, and control of spot welding electric current intensity.
- for the system responsible for shape–dimensional accuracy and for control of mechanical parameters, an optical measurement method of the shape–dimensional accuracy of the drawpiece and the subassembly being in process of the completion has been proposed. Measurement of the mechanical parameters (hardness, tensile strength and yield strength) will be performed using a nondestructive method only once—after process of the hot stamping. For this purpose the 3MA device, produced by the Franchoufer Institution [34], will be used. It was assumed that each drawpiece and each subassembly shall be controlled. Results of the quality control shall be recorded in the database together with the unique number of the drawpiece.
- for the system monitoring conditions of the machines and devices, the authors have identified the most critical problems and breakdowns occurring during production of the drawpieces in the hot stamping process, and during completion and welding of the sub-assemblies, and methods of their identification by the sensors. The most important ones include: overload of the robot drives, damaged heaters of the furnace, too low pressure in pneumatic and hydraulic circuits powering the machines and devices, failure of temperature and vibration sensors.

Acquired knowledge about the operation of the above-specified systems was suitably converted into the form allowing its usage in control algorithms used in the central control system of the production line.

## 5. Virtualization of the Production Process, Construction of “Digital Twin”

**Figure 3:**
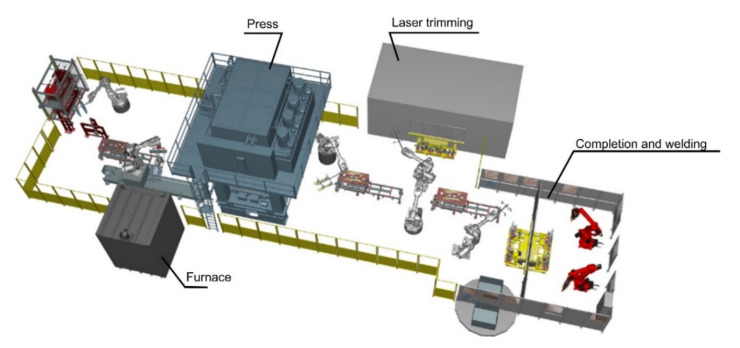
Digital model of the module for hot-stamping process.

The “digital twin” was constructed for verification of the assumptions elaborated for the automated production line [33]. It is composed of a few basic elements responsible for: technological parameters of the hot stamping process, monitoring of the machinery conditions, quality control, a system for data acquisition, and a system for drawing conclusions and taking decisions. Individual elements of the system have been implemented in the virtual PLC controller and verified using a digital model of the line in terms of the kinematics and time of operation. First, using the Siemens Tecnomatix software (Munich, Germany), a virtual model was developed to reflect the hot-stamping module; the model was equipped with digital models of the robots, furnace, press and necessary tooling. This model is shown in Figure 3. In the next step, lists of process signals, connected to the PLC controller, were elaborated. Suitable setting of states of the output signals in the PLC controller is controlling the driver elements (motors, actuators, press, robots). Each virtual object being part of the virtual twin transmits the measurement signals to the main control unit of the system, which accomplishes an appropriate algorithm for processing the signals and controlling appropriate machines. Additionally, the authors elaborated and tested the database structure serving to record the qualitative and technological parameters associated with each produced drawpiece and each subassembly. The model built in such way (digital twin) was used to test the overall control system, to test the quality control system, and to test o the system monitoring machine conditions. Results of the tests conducted on the virtual twin were used during construction of the real production line.

## 6. Conclusions

The article discusses a project of construction of the automatic production line, consistent with assumptions of the Industry 4.0 concept. Discussed here are processes of the hot stamping that are technologically advanced and carried out on automated, robotic and flexible production lines. Requirements of large automotive consortiums, which are the main recipients of the manufactured components, enforce automation of production processes, comprehensive quality control and identification of manufactured components along with access to the historical database. The database contains recorded production parameters and results of quality control for each manufactured product. The main sources of production losses are quality defects detected late, energy waste and unexpected failures of individual components of the system. The adopted assumptions will allow obtaining the control and management system of an automatic production line in accordance with concept of Industry 4.0.

## Data Availability

Not applicable.
